# *In-Situ* Characterization of Cathode Catalyst Degradation in PEM Fuel Cells

**DOI:** 10.1038/s41597-024-03662-w

**Published:** 2024-07-27

**Authors:** Patrick Schneider, Anne-Christine Scherzer, Linda Ney, Ha-Kyung Kwon, Brian D. Storey, Dietmar Gerteisen, Nada Zamel

**Affiliations:** 1 Fraunhofer Institute for Solar Energy Systems, ISE, Freiburg, Germany; 2 Toyota Research Institute, Cambridge, MA USA

**Keywords:** Fuel cells, Fuel cells

## Abstract

The composition and morphology of the cathode catalyst layer (CCL) have a significant impact on the performance and stability of polymer electrolyte membrane fuel cells (PEMFC). Understanding the primary degradation mechanism of the CCL and its influencing factors is crucial for optimizing PEMFC performance and durability. Within this work, we present comprehensive *in-situ* characterization data focused on cathode catalyst degradation. The dataset consists of 36 unique durability tests with over 4000 testing hours, including variations in the cathode ionomer to carbon ratio, platinum on carbon ratio, ionomer equivalent weight, and carbon support type. The applied accelerated stress tests were conducted with different upper potential limits and relative humidities. Characterization techniques including IV-curves, limiting current measurements, electrochemical impedance spectroscopy, and cyclic voltammetry were employed to analyse changes in performance, charge and mass transfer, and electrochemically active surface area of the catalyst. The aim of the dataset is to improve the understanding of catalyst degradation by allowing comparisons across material variations and provide practical information for other researchers in the field.

## Background & Summary

Polymer electrolyte fuel cells (PEMFC) are a promising option for clean and efficient energy conversion. However, maintaining long-term durability remains a barrier to their widespread commercialization. The initial performance and the long-term stability of a fuel cell strongly depends on the composition and morphology of the cathode catalyst layer (CCL). To optimize PEMFC durability, a comprehensive understanding of the dominating degradation mechanisms in the CCL is required. It is crucial to understand how CCL properties like the ionomer to carbon (I/C) ratio, ionomer equivalent weight (EW), carbon support type, and platinum on carbon (Pt/C) ratio affect these degradation mechanisms under different operating conditions.

Electrochemical degradation of the CCL is typically quantified in the loss of platinum (Pt) or Pt alloy catalyst by determining the change in electrochemically active surface area (ECSA) over time. Under typical operating conditions, several Pt degradation mechanisms are generally considered: Pt particle migration, Pt dissolution and re-deposition onto larger particles (Ostwald ripening), Pt ion diffusion through the CCL into the membrane, and Pt particle detachment due to carbon corrosion^1–4^. Despite widespread knowledge of these mechanisms, there is still ongoing research into what influences them and how to mitigate their impact by tailored operating strategies or material designs.

Hence, many publications have studied the impact of varying operating conditions on the long term stability of a cell^5–10^, with the main driving factors for Pt degradation being the applied cell potential and potential profile, operating temperature, and relative gas humidity (RH). While increasing humidities might increase the amount of active and hence dissolvable Pt^5,9^, higher cell potentials and temperatures accelerate degradation by increasing the rate of Pt dissolution and carbon corrosion^8,11–13^. In this context, it is especially important to understand how material properties affect the CCL morphology and hence its degradation and degradation-preventing mechanisms. Cathode parameters like ionomer to carbon ratio, platinum loading, Pt/C ratio or catalyst and support type have shown to have a strong influence on the beginning of life performance and how performance determining parameters like mass activity or charge and mass transfer develop over time.

Increasing the cathode’s ionomer content reduces the protonic resistance of the ionomer network^14–17^, but can also limit mass transport by increasing ionomer film thicknesses, leading to higher O_2_ diffusion resistances^17,18^. A higher ionomer content can additionally increase the protonically connected Pt and can accelerate the overall ECSA loss due to Pt dissolution^9^. In addition to the amount of ionomer, the ionomer’s equivalent weight (EW) is a crucial material parameter, which impacts the cell’s protonic conductivity. The EW describes the weight of polymer per mole of proton conductive SO_3_‐ groups, and represents the inverse of the ion exchange capacity (IEC)^19^. Hence, lower EWs are typically associated with higher protonic conductivities and can impact the water uptake and swelling behaviour of the ionomer^20,21^. Shimizu *et al*. reported that low conductive ionomers can additionally hinder Pt ion diffusion towards the membrane, leading to reduced Pt losses^22^.

Pt is commonly used as the primary catalyst material in PEMFCs. However, extensive research has been conducted on Pt alloys to improve the catalytic properties of the catalyst. Pt-Cobalt (PtCo) alloyed catalysts show a notable increase in mass activity compared to pure Pt catalyst^23–26^. However, during production and long term operation they suffer from strong activity losses due to cobalt leaching, which ultimately reduces the catalyst’s activity^27,28^. Independent of the Pt or Pt alloy used, the catalyst is typically present in the form of nanoparticles deposited onto a porous carbon support. These carbon support types can be categorized into low surface area carbons (LSAC), where Pt is mainly deposited on the surface of less porous carbon particles, and high surface area carbons (HSAC), where Pt is additionally present inside the pores of high porous carbon particles. The variation in Pt location influences both performance and long term stability of the cell due to the Pt accessibility by both protons and oxygen^29–32^. Alongside the location of Pt, the Pt/C ratio defines the amount of deposited Pt per carbon mass. Higher Pt/C ratios hence enable the production of high loaded catalyst layers while remaining low catalyst layer thicknesses, hence improving charge and mass transport^15,33,34^.

Although the performance and degradation trends mentioned above have been published, the corresponding data is often not or only partially available for further research by other individuals or groups. Additionally, comparisons across material variations are often only possible within specific studies. Combining datasets from different publications can be challenging due to variations in operation, testing hardware, sample preparation, and data evaluation. Since stability tests require long testing times, it is even more difficult to gather a sufficiently large and reliable dataset for data-driven analytics.

Within this work we share a comprehensive dataset of PEMFC *in-situ* characterization and durability tests, with consistent testing and production routines. The dataset contains a total of 36 unique durability tests with varying cathode I/C ratios, Pt/C ratios, Pt alloys, ionomer EWs and carbon support types, as well as variations in the operating conditions of the applied accelerated stress test. The total cumulative durability testing time represents 6 months of data. This research aims to contribute to the understanding of degradation phenomena in PEMFCs and provide valuable information for other researchers.

## Methods

Multiple variations of cathode catalyst materials were subjected to a comprehensive *in-situ* characterization including an accelerated stress test focused on degrading the cathode catalyst layer. The following cathode catalyst layer properties were varied during production: I/C ratio, Pt/C wt%, carbon support type, ionomer EW and catalyst type. Each material was subjected to a base version of an accelerated stress test over 30,000 rectangular potential cycles between 0.6 V and 0.95 V, with 3 s dwell time at each potential. As variations to this AST, each material was additionally tested with an upper potential limit of 1.15 V, while the I/C ratio and carbon support material variations were further degraded at varying relative humidities of 40%, 70% and 100%. Table 1 gives an overview of all conducted experiments, split in material and AST variations. It should be noted that test number 2 and 31 are the same dataset, as it serves as a reference in two variations. However, for better readability of the whole data collection they have been listed as separate entries. The same accounts for test numbers (3/14), (11/33), (12/18), (22/35) and (27/41), resulting in a total of 36 unique datasets.

**Table 1:** Measurements overview including variations in material composition and AST boundary conditions.

Data labels			Cathode properties						AST	
Material variation	AST variation	Cell no.	Pt load	I/C ratio	Io. EW	Pt/C ratio	Catalyst type	Support type	UPL	RH
			mg cm^−2^		g mol^−1^	%			V	%
**Ionomer to carbon ratio**	UPL 0.95 V 100% RH	**1**	0.3839	*0.5	790	50	Pt (SUP1)	HSA	0.95	100
		**2**	0.3793	*0.8	790	50	Pt (SUP1)	HSA	0.95	100
		**3**	0.381	*1.2	790	50	Pt (SUP1)	HSA	0.95	100
	UPL 0.95 V 70% RH	**4**	0.3839	*0.5	790	50	Pt (SUP1)	HSA	0.95	70
		**5**	0.3838	*0.8	790	50	Pt (SUP1)	HSA	0.95	70
		**6**	0.3827	*1.2	790	50	Pt (SUP1)	HSA	0.95	70
	UPL 0.95 V 40% RH	**7**	0.3863	*0.5	790	50	Pt (SUP1)	HSA	0.95	40
		**8**	0.381	*0.8	790	50	Pt (SUP1)	HSA	0.95	40
		**9**	0.3847	*1.2	790	50	Pt (SUP1)	HSA	0.95	40
	UPL 1.15 V 100% RH	**10**	0.3863	*0.5	790	50	Pt (SUP1)	HSA	1.15	100
		**11**	0.3816	*0.8	790	50	Pt (SUP1)	HSA	1.15	100
		**12**	0.381	*1.2	790	50	Pt (SUP1)	HSA	1.15	100
**Ionomer equivalent weight**	UPL 0.95 V 100% RH	**13**	0.3846	1.2	*720	50	Pt (SUP1)	HSA	0.95	100
		**14**	0.381	1.2	*790	50	Pt (SUP1)	HSA	0.95	100
		**15**	0.3884	1.2	*830	50	Pt (SUP1)	HSA	0.95	100
		**16**	0.3896	1.2	*980	50	Pt (SUP1)	HSA	0.95	100
	UPL 1.15 V 100% RH	**17**	0.3862	1.2	*720	50	Pt (SUP1)	HSA	1.15	100
		**18**	0.381	1.2	*790	50	Pt (SUP1)	HSA	1.15	100
		**19**	0.4162	1.2	*830	50	Pt (SUP1)	HSA	1.15	100
		**20**	0.3717	1.2	*980	50	Pt (SUP1)	HSA	1.15	100
**Platinum on carbon ratio**	UPL 0.95 V 100% RH	**21**	0.378	0.8	790	*60	Pt (SUP2)	HSA	0.95	100
		**22**	0.3995	0.8	790	*50	Pt (SUP2)	HSA	0.95	100
		**23**	0.3754	0.8	790	*40	Pt (SUP2)	HSA	0.95	100
		**24**	0.3638	0.8	790	*30	Pt (SUP2)	HSA	0.95	100
		**25**	0.4017	0.8	790	*20	Pt (SUP2)	HSA	0.95	100
	UPL 1.15 V 100% RH	**26**	0.3859	0.8	790	*60	Pt (SUP2)	HSA	1.15	100
		**27**	0.4128	0.8	790	*50	Pt (SUP2)	HSA	1.15	100
		**28**	0.3847	0.8	790	*40	Pt (SUP2)	HSA	1.15	100
		**29**	0.3755	0.8	790	*30	Pt (SUP2)	HSA	1.15	100
		**30**	0.4059	0.8	790	*20	Pt (SUP2)	HSA	1.15	100
**Platinum alloys**	UPL 0.95 V 100% RH	**31**	0.3793	0.8	790	50	*Pt (SUP1)	HSA	0.95	100
		**32**	0.3547	0.8	790	50	*PtCo (SUP1)	HSA	0.95	100
	UPL 1.15 V 100% RH	**33**	0.3816	0.8	790	50	*Pt (SUP1)	HSA	1.15	100
		**34**	0.3569	0.8	790	50	*PtCo (SUP1)	HSA	1.15	100
**Carbon support**	UPL 0.95 V 100% RH	**35**	0.4123	0.8	790	50	Pt (SUP2)	*HSA	0.95	100
		**36**	0.4142	0.8	790	50	Pt (SUP2)	*LSA	0.95	100
	UPL 0.95 V 70% RH	**37**	0.4163	0.8	790	50	Pt (SUP2)	*HSA	0.95	70
		**38**	0.4167	0.8	790	50	Pt (SUP2)	*LSA	0.95	70
	UPL 0.95 V 40% RH	**39**	0.4181	0.8	790	50	Pt (SUP2)	*HSA	0.95	40
		**40**	0.4059	0.8	790	50	Pt (SUP2)	*LSA	0.95	40
	UPL 1.15 V 100% RH	**41**	0.4128	0.8	790	50	Pt (SUP2)	*HSA	1.15	100
		**42**	0.4102	0.8	790	50	Pt (SUP2)	*LSA	1.15	100

Changing material properties are marked with a * in each variation set.

The decal transfer route has been used as a CCM production process due to its high reliability and reproducibility with different materials in this study^35^. The cathode catalyst layers were produced by screen printing on a decal substrate with a target Pt loading of 0.38 mg cm^−2^. Catalyst powders were provided by two different suppliers: Pt and PtCo catalysts on high surface area carbons (50 wt% Pt/C) provided by supplier 1 (SUP1), Pt catalysts on high surface area carbon with Pt/C ratios varying between 20 and 60 wt% provided by supplier 2 (SUP2) and Pt catalysts (50 wt% Pt/C) deposited on both high and low surface area carbon supports (SUP2). Aquivion (liquid dispersion, 25% in water, Sigma-Aldrich Chemie GmbH) with an EW of 720, 790, 830 and 980 g mol^−1^ was used as the ionomer. The ionomer and carbon content were adjusted to achieve I/C ratios of 0.5, 0.8 and 1.2. A mixture of organic solvents (50 Vol.% ethylene glycol, 50 Vol.% propylene glycol methyl ether) was used for dispersion. For the anode catalyst layers, a Umicore Elyst 0390 catalyst with 20% platinum on low surface area carbon was used to achieve a loading of 0.05 mg cm^−2^. After printing, each catalyst layer was dried in a convection dryer at 150 °C for 10 min. Each layer was afterwards transferred via hot-pressing at 180 °C and 5 MPa (referred to the printed area of 20 cm^2^) for 15 min onto a GORE SELECT membrane with 18 µm thickness. As gas diffusion layer, a Freudenberg H23C9 was used on both anode and cathode side. The ink dispersing and details of the screen-printing process are further described in a previous publication^36^. Similar screen printing procedures have also been investigated by other groups^37–40^.

All tests were carried out in a fully automated in-house developed test bench together with a “Baltic ISE qCf Liquid Cooling high amp zero gradient” test cell with 12 cm^2^ active area. The same test cell and test bench were used throughout all tests. Variations in relative gas humidity were realized by dynamically mixing dry and humidified gas streams, while the cell temperature was controlled by a Lauda Eco Silver RE1050 cryostat. For electrochemical measurements a Höcherl and Hackl PLI1206ZVSV5 electric load was used in combination with a Zahner Zennium potentiostat. During all measurements, the cells were compressed at a constant clamping pressure of 1.35 MPa.

Table 2 gives an overview of the characterization protocol applied to each material variation. A full characterization was performed at beginning of test (BoT) and end of test (EoT), while a shortened in-between characterization was performed at distinctive aging intervals. The applied protocol resulted in an approximate testing time of 120 h per material, leading to a total of over 4,000 h testing time for all 36 tests. The characterization protocol includes the methods described below. Further explanation on the displayed error bars is provided in the “Technical Validation” section.

**Table 2:** Overview of the applied test protocol, including a Break-In procedure, comprehensive beginning of test characterization, voltage cycling and shortened in-between characterization, and an end of test characterization.

	Step	Method	Test conditions		
			T_Cell_	RH_An/Ca_	p_An/Ca_ [bara]
			[°C]	[%]	
Break In	1	Galvanostatic conditioning	80	100	2
		Potentiostatic conditioning		100	2
Beginning of test characterization	2	Limiting current measurements	80	100	2
	3	IV-Curves High RH		100	2
	4	EIS H_2_/Air High RH		100	2
	5	EIS H_2_/N2 High RH		100	2
	6	IV-Curves Medium RH		70	2
	7	EIS H_2_/Air Medium RH		70	2
	8	IV-Curves Low RH		40	2
	9	EIS H_2_/Air Low RH		40	2
	10	Cyclic voltammetry		100	1
	11	Linear sweep voltammetry		100	1.5
Accelerated stress test	12	Accelerated stress test: Voltage cycling for 10, 100, 1k, 3k, 5k, 10k, 20k and 30k cycles	80	40–100	1
		Characterization in between cycling intervals: CV, EIS H_2_/Air, H_2_/N_2_ and IV-curve at high RH		100	1–2
End of test characterization	13 (2)	Limiting current measurements	80	100	2
	14 (3)	IV-Curves High RH		100	2
	15 (4)	EIS H_2_/Air High RH		100	2
	16 (5)	EIS H_2_/N_2_ High RH		100	2
	17 (6)	IV-Curves Medium RH		70	2
	18 (7)	EIS H_2_/Air Medium RH		70	2
	19 (8)	IV-Curves Low RH		40	2
	20 (9)	EIS H_2_/Air Low RH		40	2
	21 (10)	Cyclic voltammetry		100	1
	22 (11)	Linear sweep voltammetry		100	1.5
End	23	Cell shutdown	30	100	1

### Step 1 - Break-In

**Fig. 1:**
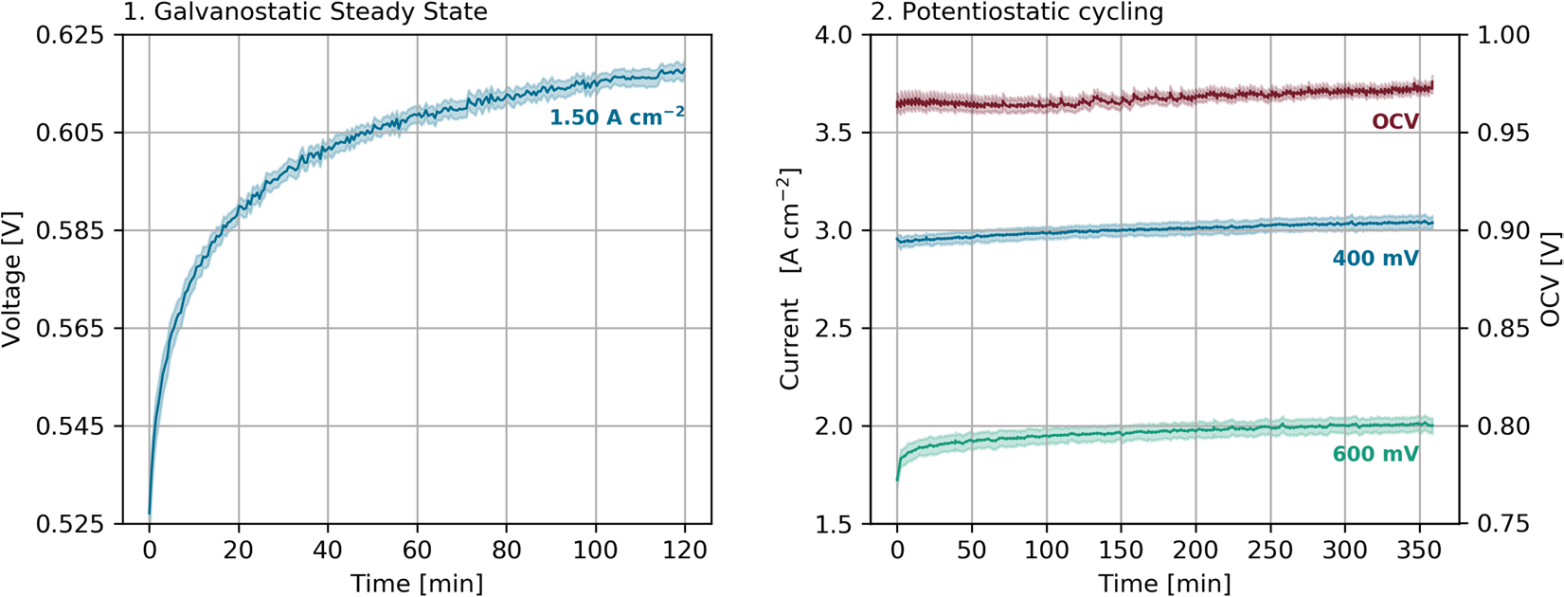
Break-In procedure for initial cell conditioning: 2 h of galvanostatic steady state operation at 1.5 A cm^−2^ (left) followed by 6 h of potentiostatic cycling between OCV, 0.6 V and 0.4 V (right). The reproducibility is indicated by transparent error bands.

As an initial conditioning procedure, each cell was first operated galvanostatic for 2 h at a constant current of 1.5 A cm^−2^, at H_2_/air (with 2 and 5 normal liter per minute (NLPM) respectively), 100% RH, 80 °C, and 2 bara gas pressure. During this conditioning step, the potential was recorded every 30 s. Afterwards, the cell was dynamically cycled between OCV (10 s), 0.6 V (60 s) and 0.4 V (60 s) for a total of 6 h in which the final 10 s in each cycle step were recorded. Figure 1 gives an overview of the acquired data during both conditioning steps. In case a cell could not reach the requested 1.5 A cm^−2^ by obtaining a voltage >0.4 V, the current density was reduced in steps of 0.2 A cm^−2^ until the voltage requirement was met.

### Step 2 - Limiting Current Measurements

**Fig. 2:**
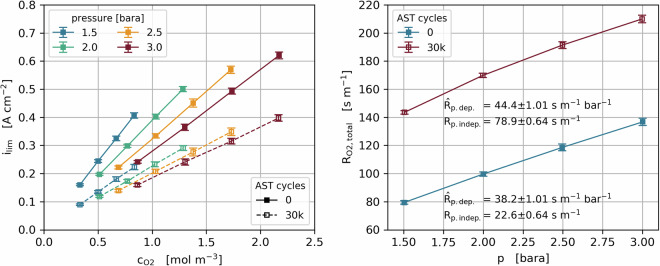
Measured limiting currents vs. O_2_ concentration and pressure (left). Calculated total mass transport resistance in each pressure and resistances split into pressure dependent and independent part (right). All values shown at both BoT and EoT of test run 34.

Limiting current measurements were carried out according to Beuscher and Baker *et al*.^41–44^ to obtain oxygen diffusion resistances. The cell was operated at 80 °C and 100% RH with 2 NLPM H_2_ on the anode and a O_2_ + N_2_ mix with a total flow of 5 NLPM on the cathode. A total of 16 limiting current values were extracted as the maximum current of voltage sweeps from 0.40 to 0.15 V, at diluted oxygen concentrations of 1, 1.5, 2 and 2.5%, and gas pressures of 1.5, 2.0, 2.5 and 3.0 bara. An exemplary data set of test run 34 is shown in Fig. 2.

The total mass transport resistance R_O2,total_ was calculated according to Eq. 1 as a function of Faradays constant F, the oxygen concentration c_O2_, and the measured limiting current i_lim_. The resistance was then averaged over all concentrations in each pressure level.

1 $${R}_{O2,{total}}=4F\frac{{c}_{o2}}{{i}_{{lim}}}$$

2 $${R}_{O2,{total}}={\hat{R}}_{p,{dep.}}\cdot p+{R}_{p,{indep}.}$$

The obtained total resistances R_O2,total_ for each pressure p were divided into a pressure dependent (molecular diffusion) and independent part (Knudsen diffusion) by linear extrapolation according to Eq. 2. The molecular diffusion is expressed as the slope of the resistance change with pressures in s m^−1^ bar^−1^.

### Step 3, 6, 8 - Current-Voltage (I-V) curves

The cell current was measured at distinctive points between open circuit voltage (OCV) and 0.2 V, at H_2_/air (with 2 and 5 NLPM respectively), 80 °C, 2 bara gas pressure and gas humidities of 40%, 70% and 100% RH.

**Fig. 3:**
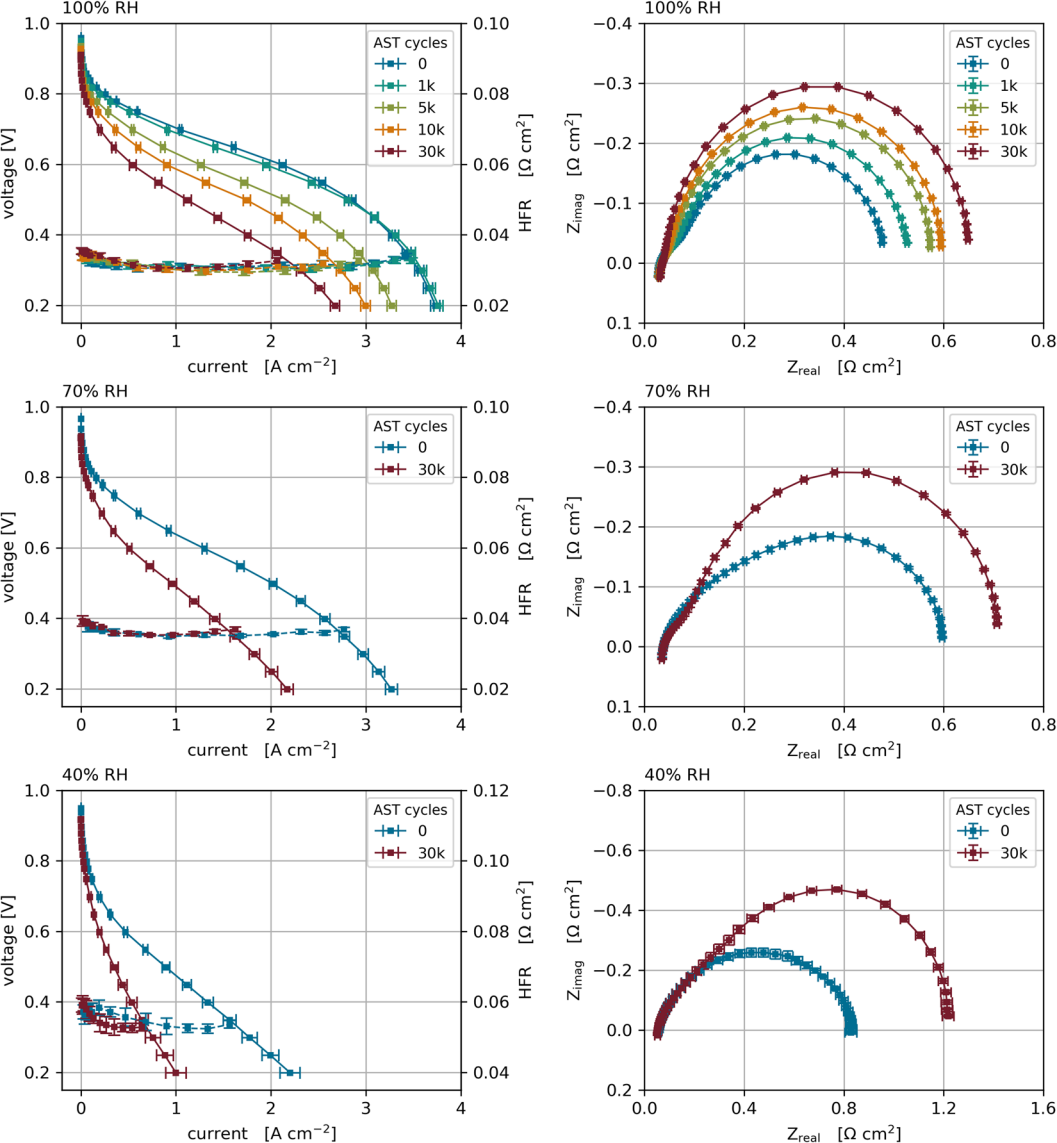
I-V-curves from BoT to EoT at 100%, 70% and 40% RH (left column) and corresponding EIS spectra at 0.1 A cm^−2^ measured from 1–10 kHz (right column). Data shown from cell 11 (I/C 0.8, EW 790 g/mol, 50 wt% Pt/C on HSA carbon, UPL 1.15 V @ 100% RH).

Each point was averaged over 30 s, after conditioning for a minimum of 5 minutes, and until a stability of <1% was reached. The high frequency resistance was measured in each operating point between 0.9 and 0.4 V, by recording a shortened impedance spectrum between 500 Hz and 10 kHz. In addition to this shortened spectrum for HFR evaluation, a full spectrum down to 1 Hz was recorded at various operating points, which is discussed in the next paragraph. The recorded HFR data was used to carry out a I-R correction of the performance data. Polarization curves were measured at BoT and EoT at all RHs, and additionally after 1k, 5k and 10k cycles at 100% RH. An exemplary data set of the recorded polarization curves as well as the respective full impedance spectra at 0.1 A cm^−2^ are shown in Fig. 3.

### Step 4, 7, 9 - Electrochemical Impedance Spectroscopy (EIS) in H_2_/air

After each polarization curve, impedance spectra were measured in H_2_/air conditions, at 2 bara, 80 °C and 100%, 70%, and 40% RH on both anode and cathode. The spectra were measured from 1 Hz – 10 kHz, at 0.1, 0.5 and 1.0 A cm^−2^ with an amplitude of 10% of the direct current. The transmission line model discussed in the H_2_/N_2_ EIS section was not employed for parameter fitting as it assumes a homogeneous electrode distribution, resulting in low accuracy when fitting EIS measured under load. To perform further analysis of the spectra, an adapted model is necessary that allows for parameter variations in the electrode along the through-plane direction.

### Step 5 - EIS in H_2_/N_2_

**Fig. 4:**
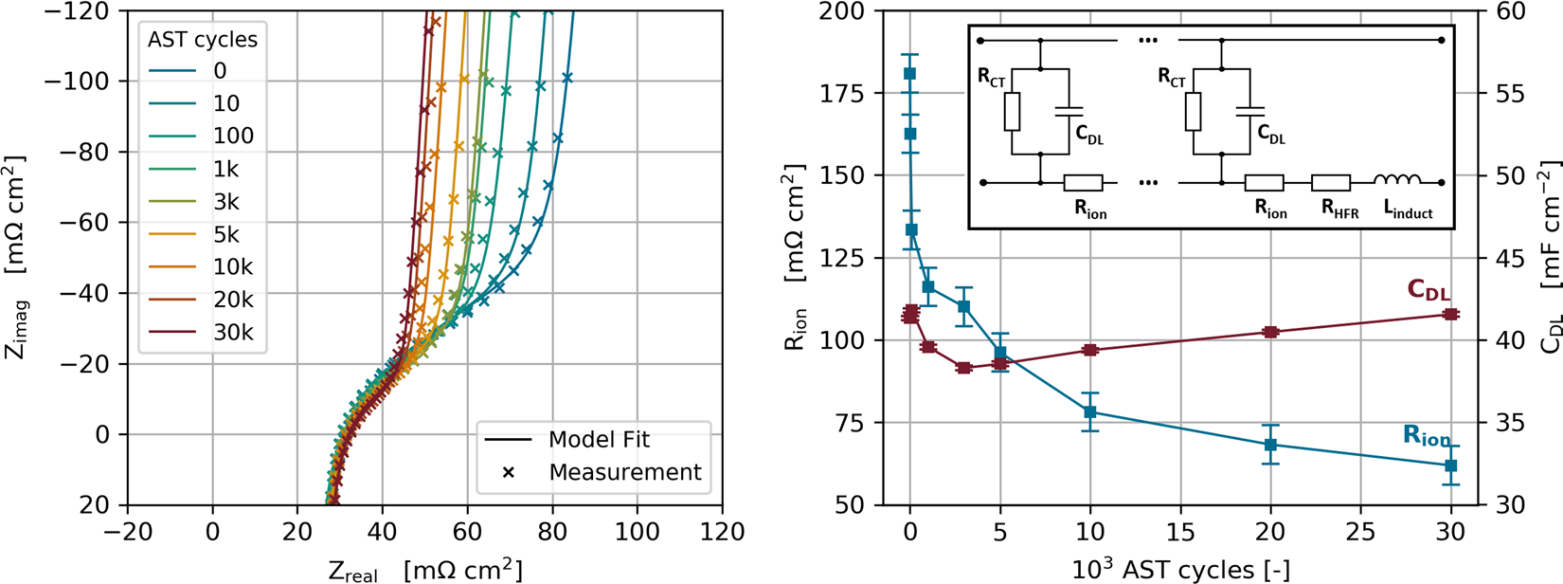
EIS spectra in H_2_/N_2_, measured from 1–10 kHz. Ionic resistance and double layer capacity evaluated by fitting with a transmission line model (equivalent circuit shown in inlay).

Impedance spectra were measured in H_2_/N_2_ at 200 and 450 mV with a 10 mV amplitude, at 80 °C, atmospheric pressure and 100% RH on both anode and cathode. A transmission line model adapted from Makharia *et al*.^45^ was used to evaluate the spectra, which accounts for double layer capacity C_DL_, protonic catalyst layer resistance R_ion_, high frequency resistance R_HFR_, charge transfer resistance R_CT_ and hardware inductivity L_induct._. To achieve better fit accuracy, the capacitors have been replaced with constant phase elements, introducing the exponent φ. Figure 4 shows an example data set of the fitted impedance data and the corresponding parameters.

The resulting equivalent circuit is shown in the inset of Fig. 4. The total impedance of the circuit can be described by Eqs. 3, 4:

3 $$Z(\omega )=\sqrt{{R}_{{ion}}{Z}_{{RC}}}\,\coth \,\left(\sqrt{\frac{{R}_{{ion}}}{{Z}_{{RC}}}}\right)+{R}_{{HFR}}+j\omega L$$

4 $${Z}_{{RC}}=\frac{{R}_{{CT}}}{1+{R}_{{CT}}{(j\omega )}^{\varphi }{C}_{{DL}}}$$

### Step 10, 11 - Cyclic Voltammetry (CV) and Linear Sweep Voltammetry (LSV)

**Fig. 5:**
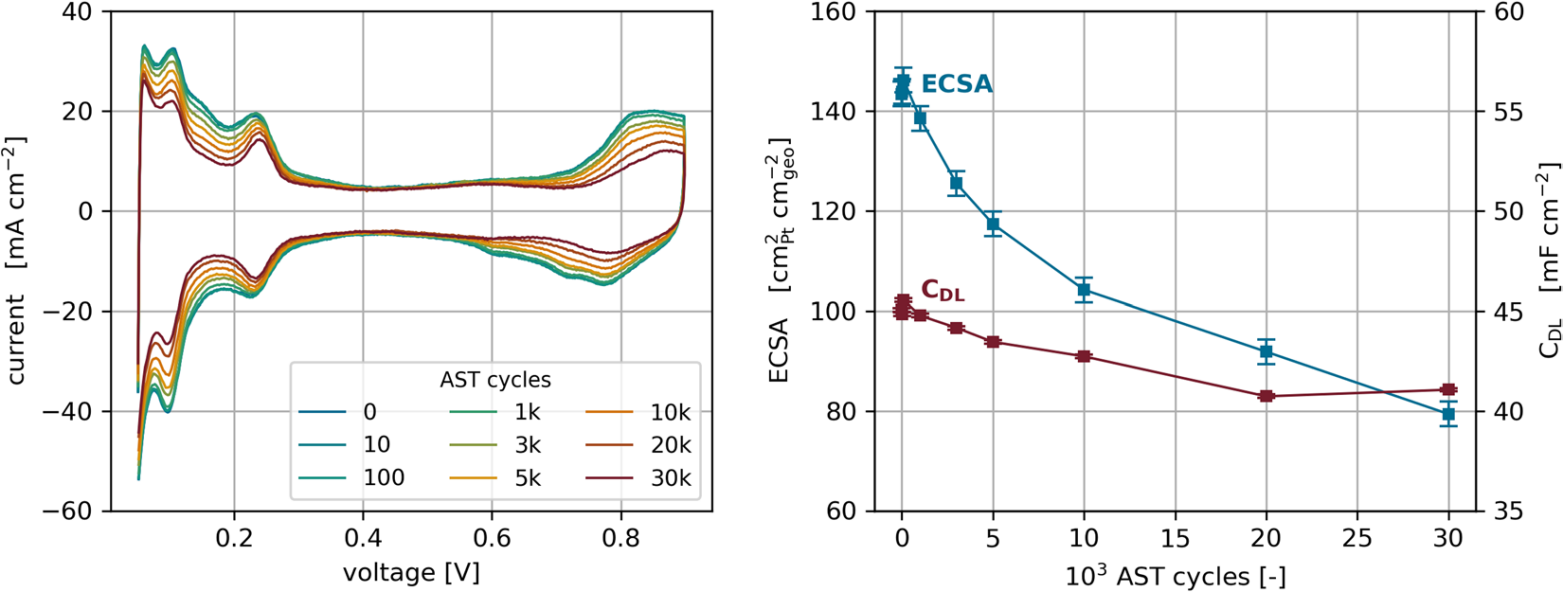
Cyclic voltammetry and evaluated ECSA and double layer capacity over time for cell 2 (I/C 0.8, EW 790 g mol^−1^, 50 wt% Pt/C on HSA carbon, UPL 0.95 V @ 100% RH).

For the CV, the cell was cycled 5 times between 0.05 and 0.95 V at 100 mV s^−1^ scan rate, with 1 NLPM H_2_ on the anode and no flow in the cathode. The measurement was performed at 80 °C and 100% RH at atmospheric pressure. Prior to the voltage cycling, the cell was conditioned for 15 minutes in the mentioned conditions. Afterwards, the ECSA was obtained by averaging the H_2_ adsorption and desorption charge, and the double layer capacity was evaluated at the minimum in the region between 0.3 and 0.6 V^46^. CVs were conducted in each aging step from 0 to 30k cycles. Figure 5 displays an exemplary CV dataset of test run 2. The LSV was performed in the same conditions, only with 1 NLPM N_2_ on the cathode, and a potential sweep from 0.1 to 0.5 V with 1 mV s^−1^ scan rate. The H_2_ crossover current was evaluated as the current at 200 mV in a linear fit of the LSV curve between 0.3 and 0.5 V^47^.

### Step 12 - Accelerated Stress Test (AST)

**Fig. 6:**
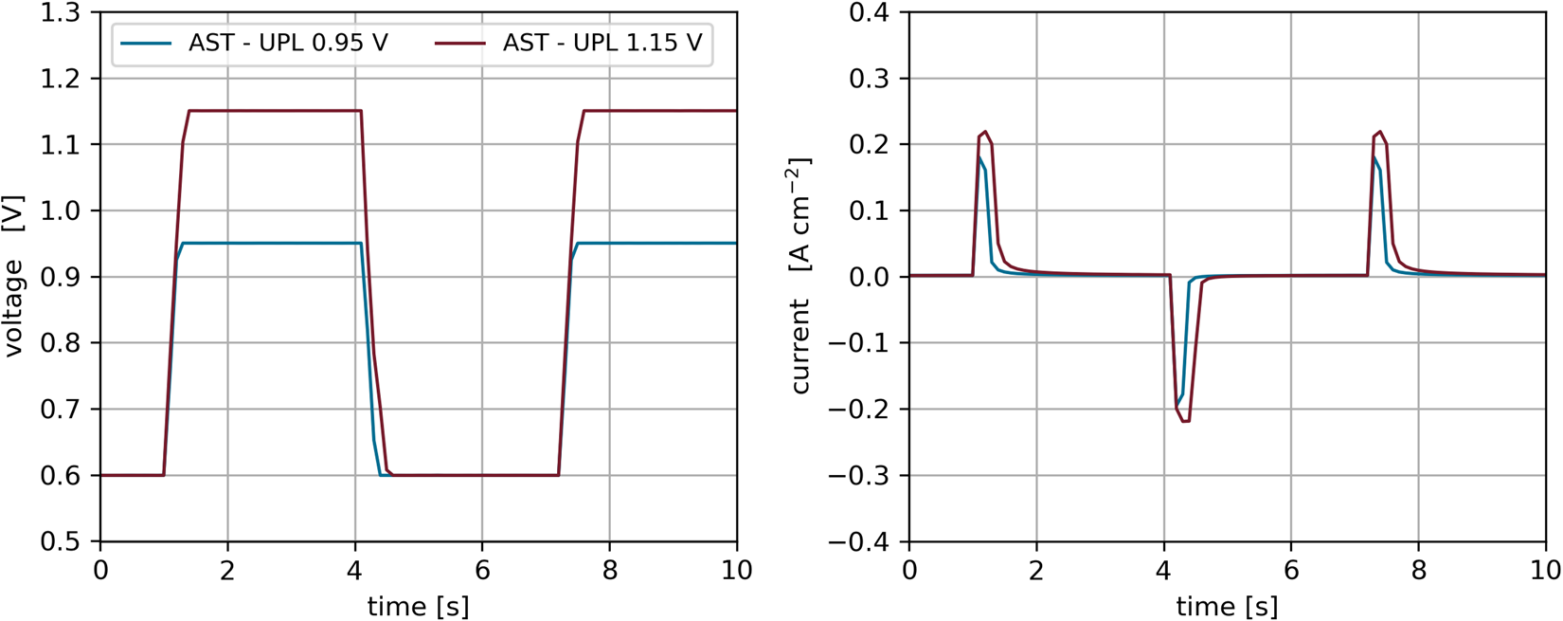
Applied accelerated stress test, voltage cycling between 0.6 and 0.95 V and 1.15 V with 3 s dwell time at each potential (left) and cell current response (right).

The catalyst degradation was performed according to the protocol proposed by the US Department of Energy (DoE), where the cell is operated at 80 °C, in H_2_/N_2_ atmosphere and cycled between 0.6 and a fixed upper potential limit (UPL) for 30,000 cycles, with 3 s dwell time at each potential and a fixed slope time of 0.25 s between potentials. For all material variations, measurements were conducted with an UPL of 0.95 V and 1.15 V. For the I/C ratio and carbon support variation the AST was additionally performed at 100%, 70% and 40% RH. Current and voltage data was recorded at 10 Hz during the AST as shown in Fig. 6.

## Data Records

The data of all test runs stated in Table 1 is stored in a figshare repository and can be accessed through the link in the ref. ^48^. The storage is structured in a sub-folder system, with the main sub-folder level representing the test runs (cells 1–42, as in Table 1), inheriting sub-folders for the test method (steps 1–12, as in Table 2) and finally subfolders for the degradation interval (0–30k AST cycles). At the end of each sub folder path the corresponding data is stored in form of a csv-file, where some files contain multiple rows and columns (e.g. raw CV or impedance data), while others consist of a single evaluated parameter value (e.g. ECSA and protonic resistance). Table 3 gives an overview of every available data label (csv file) for each applied characterization method and a description of the data columns each file contains. The last column indicates at which AST cycle step (0–30k) each characterization data is available.

**Table 3:** Overview of each data label (csv file) and the inheriting data columns in each applied characterization method.

Step	Method	Available data label (*.csv)	Description	Available data columns	Cycle step
1	Break-In	BreakIn_data	Potentiostatic/Galvanostatic data during cell conditioning	Time [min]	0
				Voltage [V]	
				Current [A cm^−2^]	
2	Limiting current measurements	iLim_data	limiting currents extracted from voltage sweeps in each pressure and O_2_ concentration	Current [A cm^−2^]	0, 30k
				O_2_ conc. [mol m^−3^]	
				Pressure [bara]	
		R_total_p	Total mass transport resistance vs. pressure	Total MTR [s m^−1^]	
				Pressure [bara]	
		R_p_indep	pressure independent resistance part	Resistance [s m^−1^]	
		R_p_dep	pressure dependent resistance part	Res. slope [s m^−1^ bara^−1^]	
3	IV-Curves: High RH	IV_data	Averaged data from current/voltage curves	Voltage [V]	0, 1k, 5k, 10k, 30k
				Current [A cm^−2^]	
		IV_data_IR_corr	Averaged current/voltage curves corrected by respective HFR value	Voltage [V]	
				Current [A cm^−2^]	
		HFR_data	HFR in each U/I curve operating point	Current [A cm^−2^]	
				HFR [mΩ cm^2^]	
		EIS_data_HF	high frequency EIS spectra from 0.5–10 kHz	Frequency [Hz]	
				Imp. real [mΩ cm^2^]	
				Imp. imag. [mΩ cm^2^]	
				Current [A cm^−2^]	
4	EIS H_2_/Air	EIS_data_raw	raw data EIS spectra data from 1–10 kHz	Frequency [Hz]	0, 1k, 5k, 10k, 30k
				Imp. real [mΩ cm^2^]	
				Imp. imag. [mΩ cm^2^]	
5	EIS H_2_/N_2_: High RH	EIS_data_raw	raw data EIS spectra data from 1–10 kHz	Frequency [Hz]	0, 10, 100, 1k, 3k, 5k, 10k, 20k, 30k
				Imp. real [mΩ cm^2^]	
				Imp. imag. [mΩ cm^2^]	
		R_ion	protonic catalyst layer resistance (fitted from TLM)	protonic catalyst layer resistance [mΩ cm2]	
		HFR	high frequency resistance (fitted from TLM)	HFR [mΩ cm^2^]	
		C_DL	double layer capacity (fitted from TLM)	DLC [mF cm^−2^]	
6–9	IV-Curves, EIS H2/Air: Med./Low RH	See step 3–4			0, 30k
10	CV	CV_data_raw	raw data from cyclic voltammetry (5 cycles, 0.05–0.9 V)	Time [s]	0, 10, 100, 1k, 3k, 5k, 10k, 20k, 30k
				Voltage [V]	
				Current [mA cm^−2^]	
		CV_cycle_corr	last CV cycle, crossover and shorting current corrected	Voltage [V]	
				Current [mA cm^−2^]	
		ECSA_mean	ECSA averaged over all CV cycles	ECSA [cm_Pt_^2^ cm_geo_^−2^]	
		C_DL_mean	Double layer capacity averaged over all CV cycles	DLC [mF cm^−2^]	
11	LSV	LSV_data_raw	raw data from linear sweep voltammetry (0.1–0.5 V)	Time [s]	0, 30k
				Voltage [V]	
				Current [mA cm^−2^]	
		I_xover	crossover current fitted from linear sweep	Crossover current [mA cm2]	
12	AST	pot_cycles	voltage cycling data over time	Time [s]	10, 100, 1k, 3k, 5k, 10k, 20k, 30k
				Voltage [V]	
				Current [A cm^−2^]	

The last column indicates the data availability at each cycle step from 0–30k AST cycles.

## Technical Validation

All measurements have been conducted on the same testbench using the same testing equipment and cell hardware throughout the duration of the project. The measurement protocol was performed in a fully automated way to assure full reproducibility and comparability between tested samples. Prior to the project, all sensors and testing hardware have been calibrated and are subject to the following errors:

- Temperatures: +/−0.2 K
- Pressures: +/−25 mbar
- Current/Voltage (Electric load): +/−0.1% of current/voltage set value
- Current/Voltage (Potentiostat): +/−0.05% of current set value, +/−50 µV

During the duration of the measurements, no changes were made to the test hardware. Additionally purchased materials like gas diffusion layers and membranes have been taken from the same production batch throughout all measurements.

To ensure the reliability and reproducibility of our dataset, error bars are included in the displayed characterization plots. These error bars represent the standard deviation calculated from the BoT characterization data of four cells (cell 36, 38, 40, 42) with identical initial material specifications. This deviation hence accounts for the reproducibility of both the MEA production process and the *in-situ* testing protocols.

## Usage Notes

It should be noted that there are some data gaps in the EIS measurements. In certain cases, galvanostatic EIS measurements could not be conducted at all operating points due to the cell’s degraded state preventing higher current densities from being reached. As a result, these subfolders only include impedance data where the desired conditions could be met. Additionally, for cell 9, there is a data gap with all EIS-related data missing due to a malfunction in the potentiostat. The rest of this test run was not affected by this issue.

## Data Availability

All recorded data has been processed and evaluated with Python based scripts. Exemplary evaluation codes are available in the uploaded repository, providing information on how specific parameters have been evaluated from the respective raw data files.
